# Effect of High-Temperature Stress on Plant Physiological Traits and Mycorrhizal Symbiosis in Maize Plants

**DOI:** 10.3390/jof7100867

**Published:** 2021-10-16

**Authors:** Sonal Mathur, Richa Agnihotri, Mahaveer P. Sharma, Vangimalla R. Reddy, Anjana Jajoo

**Affiliations:** 1 Photosynthesis Laboratory, School of Life Sciences, Devi Ahilya University, Indore 452001, India; anjanajajoo@hotmail.com; 2 Adaptive Cropping Systems Laboratory, USDA-ARS, Beltsville Agricultural Research Center, Beltsville, MD 20750, USA; vr.reddy@usda.gov; 3 ICAR-Indian Institute of Soybean Research, Indore 452001, India; agnihotri.ri7@gmail.com (R.A.); mahaveer620@gmail.com (M.P.S.); 4 School of Biotechnology, Devi Ahilya University, Indore 452001, India

**Keywords:** arbuscular mycorrhizal fungi, fatty acid biomarkers, high temperature, maize (*Zea mays* L.), photosynthesis, PSI, PSII

## Abstract

Increasing high temperature (HT) has a deleterious effect on plant growth. Earlier works reported the protective role of arbuscular mycorrhizal fungi (AMF) under stress conditions, particularly influencing the physiological parameters. However, the protective role of AMF under high-temperature stress examining physiological parameters with characteristic phospholipid fatty acids (PLFA) of soil microbial communities including AMF has not been studied. This work aims to study how high-temperature stress affects photosynthetic and below-ground traits in maize plants with and without AMF. Photosynthetic parameters like quantum yield of photosystem (PS) II, PSI, electron transport, and fractions of open reaction centers decreased in HT exposed plants, but recovered in AMF + HT plants. AMF + HT plants had significantly higher AM-signature 16:1ω5cis neutral lipid fatty acid (NLFA), spore density in soil, and root colonization with lower lipid peroxidation than non-mycorrhizal HT plants. As a result, enriched plants had more active living biomass, which improved photosynthetic efficiency when exposed to heat. This study provides an understanding of how AM-mediated plants can tolerate high temperatures while maintaining the stability of their photosynthetic apparatus. This is the first study to combine above- and below-ground traits, which could lead to a new understanding of plant and rhizosphere stress.

## 1. Introduction

Maize (*Zea mays* L.) is one of the important and widely grown commercial crops of the world. Maize is known for its adaptability to varying climatic conditions [1]. Higher temperatures (HT) (35 °C and above) affect the vegetative and reproductive growth of maize, from germination to grain filling [2]. High-temperature stress-induced responses in plants include modifications in the photosynthetic machinery, organizational changes in cellular structures to maintain membrane functioning, and stomatal closure to limit transpirational water loss [3]. A slight increase in temperature can have a negative impact on growing crops, grain filling, and ultimately yield [4]. Jagdish et al. [5] reported that heat stress such as short term HT, heat waves, or long term high temperature, have a detrimental effect on plant growth and yield [5].

Plants depend on rhizosphere microflora to protect them from a variety of environmental stresses [6]. Arbuscular mycorrhizal fungi (AMF) are the most prevalent among them, forming a symbiotic association with the roots of almost 80% of terrestrial plants [7]. AMF improves plant growth under stressful conditions by modulating multiple communication events leading to enhanced photosynthetic rate [8]. AMF augments plant stress tolerance by increasing nutrient levels, improving stomatal regulation, water use efficiency to reduce oxidative damage, modifying hormonal balance and osmotic adjustments [9,10,11]. The inoculation with AMF enables host plants to cope up with stressful conditions such as salinity, drought, and heavy metals [12,13,14]. 

Photosynthesis is considered one of the critical processes that decouple under various abiotic stresses. When compared to other photosynthesis protein complexes, high-temperature stress causes the most damage to photosystem II (PSII) and reduces the leaf photosynthetic capacity [3,15]. 

Studies evaluating the role of AMF in the mitigation of high-temperature stress on plants are often based on the effects on plant physiological traits. However, soil microbial communities are also an important factor for determining plant performance and may provide important insights into AMF mediated stress mitigation. [16,17]. The lipid profiling of signature markers (e.g., 16:1ω5cis phospholipid fatty acid (PLFA) and neutral lipid fatty acids (NLFA) in soil and roots) provides a detailed account of the alterations in the microbial community due to the stress caused by elevated temperature [18,19,20] and is used as a stress indicator [21]. The ratios of cyclopropyl to monoenoic fatty acids, as well as Gram-positive to Gram-negative bacteria, are PLFA stress indicators that explain temperature impacts. The trans/cis stress ratio and the Gram-negative stress ratio are two physiological stress indicators. The Gram-positive/Gram-negative ratio is an indicator of energy limitation [22]. 

In our previous study, we demonstrated that AMF ameliorates the effect of high-temperature stress in maize plants, particularly on PSII [23]. Since high temperature is a global problem and maize is one of the important crops, it becomes imperative to carry out an in-depth study with AMF enriched soil and its implications on high temperature. Thus, to focus the problem and to understand the role of AMF formation in plants exposed to high temperature we tested a hypothesis for how AM-mediated plants are modulated by high temperature and consequently, how it influences the photosynthetic (PSI, PSII) efficiency and soil PLFA microbial communities of fatty acids biomarkers. We present the hypothesis that when AM-enriched plants are exposed to high temperatures, the presence of their active hyphal biomass facilitates the plants to have better water and nutrient uptake, photosynthetic efficiency and protects plants from oxidative damage. We also tested whether the plants exposed to high temperatures depict any alteration in AMF formation (root colonization, spore density and lipid biomarkers), plant photosynthetic parameters and shift in soil PLFA microbial communities as stress perturbations? 

Thus, combining photosynthetic parameters with signature fatty acid biomarkers in relation to high-temperature stress may help unravel the role of AMF and the belowground system in adjusting the plants from stressful conditions. Information from this study will pave the way to utilize AMF as a stress ameliorator for improving abiotic stress tolerance in crop plants. 

## 2. Materials and Methods

### 2.1. Plant Material, AMF Inoculum

Maize (*Zea mays* L.; cultivar Navjot) was used as the experimental plant. Rhizosphere soil from the long-term soybean-based farming system managed at ICAR-Indian Institute of Soybean Research (IISR) Indore; was collected and sieved using the wet sieving decanting method [24]. A mixed starter culture of native AMF (which mainly comprised of *Rhizophagus irregulariae*, *Funneliformis mosseae*, and other *Glomus* species, procured from ICAR-IISR, Indore) was prepared as per the method of Sharma et al. [25] The starter culture was further raised on maize for 16 weeks using sterilized soil: sand mix (3:1 ratio) and this was amended with field yard manure (FYM) (5:1) and used for enrichment. To enrich the soil for AMF formation, the starter culture was mixed with field soil and raised on maize for several weeks as per the method of Mathur et al. [23]. The standard method of strain preparation was followed. Experiments were carried in unsterilized soil having 2–3 spores/10 g of soil. For the enriched pots, the soils were prepared by adding 50 g of mixed AMF inoculum consisting of 2500 spores. The inoculum was multiplied by growing maize for one cycle. The final spore count of the enriched soil was 20–25 spores/10 g. The experiment was conducted in two sets. The AM spore density of AMF enriched soil ranged from 20–25 spores/10 g soil. Microcosm (pot) trial) was conducted in Vertisols soil [pH 7.5 (1:2.5 soil water ratio), organic carbon (0.5%), Olsen P (6.2 mg/kg), mineral N (6.4 mg/kg)] with and without AMF.

The experiment was conducted in pots (black gusseted polythene bags with 25 kg capacity, holes for drainage) filled with medium black cotton soil previously mixed with well-decomposed farmyard manure (FYM) in a ratio of 5:1, soil to FYM). All pots received 5–7 maize seeds, which were later thinned to three plants per pot.

### 2.2. Growing Conditions and Experimental Design

Plants were watered daily (~1.5 lit in each pot) to avoid any type of water stress due to high temperatures. India is a tropical country, where the average temperature for most of the days during summer is between 40 to 44 °C. The maximum temperature at the time of experiments was found to be, 43–44 °C (month of May) (Indore, 22°44′ N), (www.acuweather.com, (17–28th May 2018) ICAR-IISR, Indore, India). For HT stress, right from the sowing till harvesting, pots were kept on the open terrace where the plants faced a maximum day temperature of 43 °C (±0.2 °C), while the relative humidity was ±28%

The experiment design was laid out in a completely randomized design in three replications (three pots for each treatment, and each pot had 3 plants) with the following treatments:

Control = maize plants were grown in normal soil and plants were not subjected to high temperature.

AMF = maize plants were grown in AMF enriched soil and plants were not subjected to high temperature.

HT = maize plants were grown in normal soil and experienced higher temperatures (ambient temperature during summer 43 °C).

AMF + HT = maize plants were grown in AMF enriched soil and experienced high-temperature stress.

The plants were harvested after 100 days of sowing at maturity. Rhizosphere soil samples were collected for high temperature exposure and control plants by uprooting the plants and gently shaking off the soil adhering to the roots. One part of the soil samples was kept at −20 °C for PLFA analysis and the rest was air-dried, sieved, and processed for the estimation of AM spore density.

### 2.3. Measurement of Total Chlorophyll Content

Total chlorophyll (Chl) content (in SPAD units) in leaves was measured using at leaf SPAD chlorophyll meter (FT Green LLC, Wilmington, DE, USA) according to Zhu et al. [26]. All the measurements were performed between 11:00 AM to12:00 PM under natural sunlight.

### 2.4. Chlorophyll a Fluorescence Measurement

Quantum yield of PSI and PSII were measured with Dual-PAM-100 system (Heinz Walz, GmbH, Effeltrich, Germany) according to Pfündel et al. [27]. Plants were kept in dark for 30 min before measurements. A weak modulated light (12 μE) was given to get minimal fluorescence (F_o_), followed by actinic light (55 μE), and saturating pulse (6000 μE) to obtain maximum fluorescence (F_m_). Leaves were exposed to saturating pulse (one pulse per min) for 10 min to obtain steady-state fluorescence. Details of various chlorophyll (Chl) *a* fluorescence parameters are presented in Table 1. Y(II), Y(NPQ), Y(NO), qL (Table 1) were calculated as per Kramer et al. [28]. The quantum yields of PSI and PSII were measured by saturating pulses during the process of the slow induction curve. Parameters were evaluated automatically according to the methods of Kramer et al. [28] and Klughammer and Schreiber [29] (Table 1):

YII = (F_m_′ − F)/F_m_′, YNPQ = F/F_m_′ − F/F_m_, YNO = F/F_m_

YI = (P_m_′ − P)/P_m_, YND = (P − Po)/P_m_, YNA = (P_m_ − P_m_′)/P_m_

### 2.5. Measurement of Malondialdehyde (MDA) Content

MDA content was measured by estimating thiobarbituric acid reactive substances (TBARS) using the method described by Zhang and Qu [33]. The absorbance was read at 532, 600, and 450 nm. The MDA content was calculated using the formula:

6.45 × (*A*_532_ − *A*_600_) − 0.56 × *A*_450_.

### 2.6. AMF Root Colonization and Spore Density

Fresh roots were carefully washed under running tap water and then cut into 1 cm pieces. A total of 100 segments were measured for each treatment. Mycorrhizal colonization in roots was examined microscopically (at 40× and 100× magnification) after digesting and clearing the roots in KOH and staining with trypan blue (0.05% in lactoglycerol) [34]. The frequency distribution method of Biermann and Linderman [35] was used for studying colonization in stained root segments based on hyphal infection, the number of vesicles and arbuscules present in the individual segment. 

### 2.7. Quantification of Signature Fatty Acids in Soil

Phospholipid fatty acid analysis (PLFA) was performed following the high throughput method [19,36]. The four steps viz., drying, extraction, lipid separation, and transesterification were performed consecutively. Bligh dyer extraction of about 1.5 g of freeze-dried soil sample was performed, following which the separation and extraction of lipids were performed using a 96 well soil phase extraction (SPE) column. The neutral lipids and phospholipids were eluted, respectively, using chloroform and methanol: chloroform: H_2_O (5:5:1) fraction. After transesterification, the extracted fatty acids were dissolved in hexane and stored in 2 mL vials for subsequent analysis using a gas chromatograph (Agilent 7890A Agilent Technologies, Wilmington, DE, USA). The samples were analyzed using the flame ionization detector on GC controlled with Agilent Chemstation (MIDI Inc., Newark, DE, USA). Agilent column (HP-Ultra 2 column, 25 m long × 0.2 mm internal diameter × 0.33 μm film thickness) was used to separate FAMEs and run the program as per the method in [36]. For the identification of FAME profiles, the MIDI PLFAD1 calibration mix and peak naming table (MIDI, Inc., Newark, DE, USA) was used. The signature fatty acid biomarkers for microbial groups were assessed and expressed as PLFA nanomoles g^−1^ soil. 

The PLFAs were summed into biomarker categories as follows: Gram-positive bacteria, iso and anteiso saturated branched fatty acids; Gram-negative bacteria, mono-unsaturated fatty acids, and cyclopropyl 17:0 and 19:0, actinomycetes, 10-methyl fatty acids, fungi, 18:2 ω6cis, and arbuscular mycorrhizal fungi, 16:1 ω5 cis fatty acids [37]. The ratio of fungi/bacteria (total fungal lipids/total bacterial lipids) and Gram-positive/Gram-negative (trans/cis stress ratio or 16:1ω7t/16:1ω7c) were also determined. 

### 2.8. Statistical Analysis

Graphs and data for chlorophyll fluorescence were analyzed by using Origin Pro8. The total chlorophyll and MDA content were analyzed using GraphPad Prism 5.01 Software (software, Inc., San Diego California USA.). Significance was determined at *p* < 0.01 (* *p* < 0.05, ** *p* < 0.01 and *** *p* < 0.001) and the results are expressed as mean values and standard deviation (SD). The data were statistically analyzed using the one-way analysis of variance (ANOVA) carried out with the COSTAT software [38]. To compare the variances between means, a least significant difference (LSD) through Duncan’s multiple range test (DMRT) at a significance level of *p* < 0.05 was used. The physiological, microscopic, and biochemical parameters were subjected to principal component analysis (PCA) using R studio version 4.0.0, Boston, Massachusetts [39,40,41] to identify the pattern of variation between parameters, and differences among treatments. All the experiments were carried out with three replicates for each treatment.

## 3. Results 

### 3.1. Total Chlorophyll Content Measurement

AMF-colonized plants had higher total chlorophyll (Chl) content (Table 2) as compared to control plants. Due to temperature stress, water availability was reduced which drastically decreased the total chlorophyll content in high temperature exposed maize plants (Table 2). AMF + HT plants performed better and recovered total chlorophyll content as compared to high temperature exposed plants (Table 2).

### 3.2. Chlorophyll (Chl) a Fluorescence Measurements

**Figure 1:**
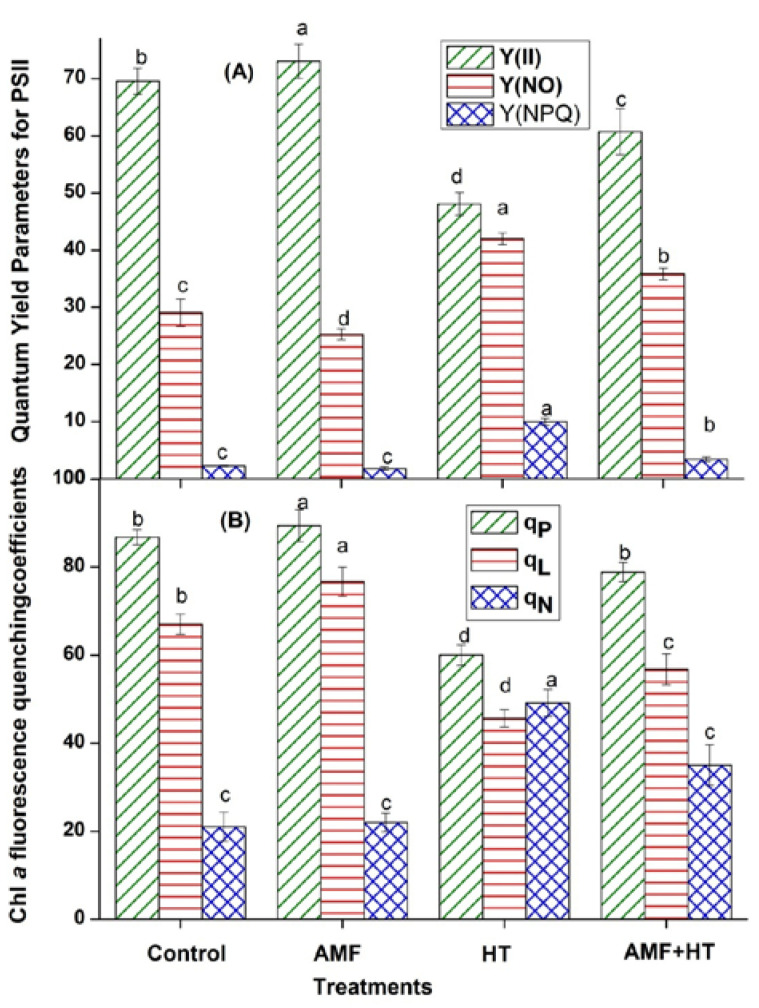
Quantum yields of energy conversion in PSII in control, AMF, high temperature (HT), AMF + HT for (**A**) Y(II) = quantum yield of PSII, Y(NO) = the yield of non-regulated energy dissipation, Y(NPQ) = the yield of regulated energy dissipation, (**B**) Chlorophyll fluorescence quenching coefficients (q_P_, q_L_, q_N_) in maize leaves under high temperature. The data are the mean values of three replicates ± standard deviation, treatment means followed with different letters vary significantly at *p* = 0.05 in compliance with Fisher least significant differences (LSD) and Duncan multiple range test (DMRT) for multiple comparisons.

Parameters of Chl fluorescence are powerful indicators for the functioning of the photosynthetic apparatus under stress conditions. In PSII, light energy partitioning can be assessed by Chl *a* fluorescence yield parameters using the saturation pulse (SP) method. Chlorophyll *a* fluorescence kinetics significantly differed among the treatments. The maximum quantum yield of PSII (YII) decreased considerably with high temperature exposure. In contrast, Y(NO) and Y(NPQ) increased in high temperature exposed maize plants (Figure 1A). AMF plants had maximum Y(II) while Y(NO) and Y(NPQ) were found to be minimum (Figure 1A). All these parameters were recovered in AMF + HT maize plants (Figure 1A).

In this study, most of the PSII centers were open in AMF plants, that is, q_L_ increased for AMF while they were closed in high temperature exposed maize plants (Figure 1B). In AMF + HT plants, more centers were in open form as compared to HT plants. q_P_ increased in AMF and control plants while decreased drastically in HT plants (Figure 1B). q_N_ was lower in control and AMF plants while increased in high temperature exposed plants (Figure 1B). These ratios were moderate in AMF + HT plants thus showing protection of the plants from severe temperature stress (Figure 1B). 

**Figure 2:**
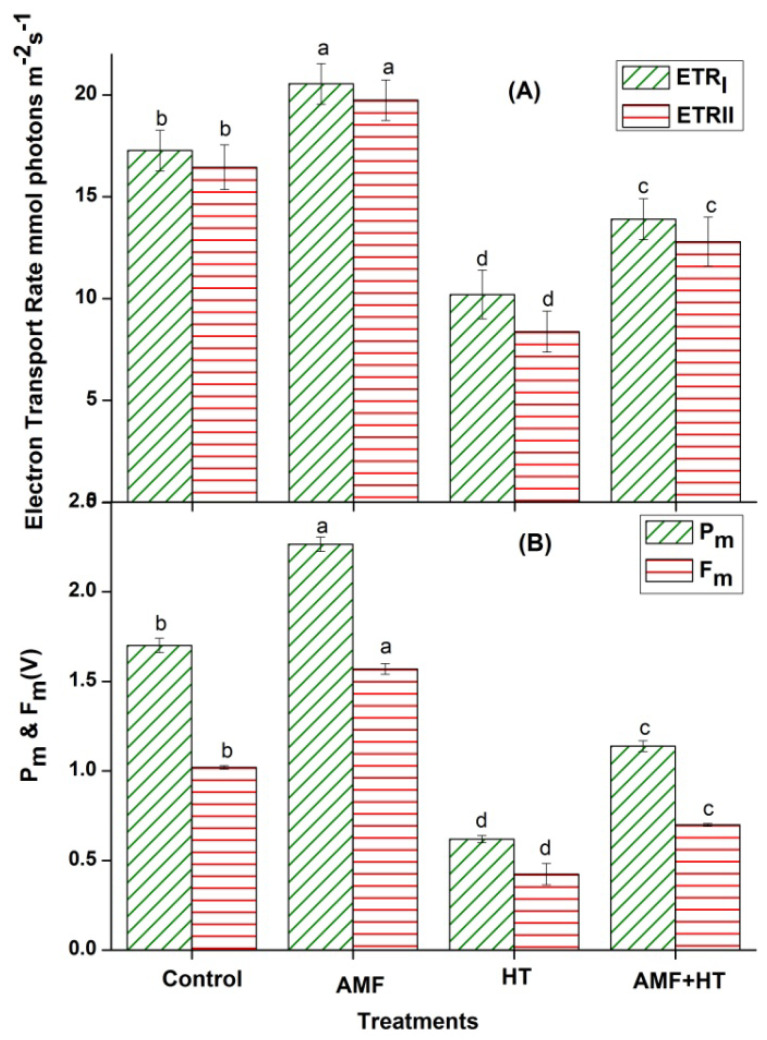
Response of (**A**) electron transport rates in PSI (ETR_I_) and PSII (ETR_II_), (**B**) maximal change in P700 (Pm) and maximum fluorescence (F_m_) signal, assessed in maize leaves. The data are the mean values of three replicates ± standard deviation, treatment means followed with different letters vary significantly at *p* = 0.05 in compliance with Fisher least significant differences (LSD) and Duncan multiple range test (DMRT) for multiple comparisons.

As compared to control plants, the highest electron transport rates were observed in AMF plants, while these rates decreased drastically in HT plants (Figure 2A). AMF + HT plants showed a higher ratio for electron transport rates (Figure 2A). P_m_ is considered one of the best indicators for PSI activity and represents the total amount of photo-oxidizable P700. AMF inoculated plants without high temperature exposure showed significantly higher P_m_ and F_m_ followed by control and AMF + HT. P_m_ and F_m_ showed a significant decrease in HT plants while comparatively higher values in AMF + HT indicate a possible restoration in plants resulting from the alleviation of HT stress by AMF (Figure 2B). The decrease in F_m_ and P_m_ was accompanied by a declined electron transport rate of PSII and PSI (ETR_II_ and ETR_I_) for HT plants as well. 

**Figure 3:**
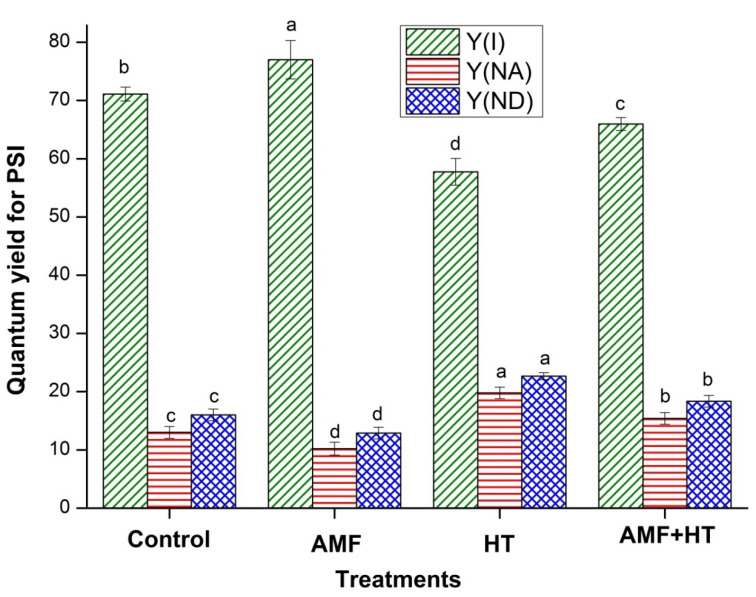
Quantum yields of energy conversion in PSI in control, AMF, high temperature (HT), AMF + HT maize leaves under high temperature, for Y(I) = quantum yield of PSI, Y(NA) = the quantum yield of non-photochemical energy dissipation caused by acceptor-side limitation, Y(ND) = is the quantum yield of non-photochemical energy dissipation caused by donor side limitation. The data are the mean values of three replicates ± standard deviation, treatment means followed with different letters vary significantly at *p* = 0.05 in compliance with Fisher least significant differences (LSD) and Duncan multiple range test (DMRT) for multiple comparisons.

Figure 3 represents the analysis of quantum efficiency of PSI Y(I), Y(NA), and Y(ND). Y(I) was found to be maximum in AMF plants while minimum in HT plants. Further, a remarkable enhancement in Y(NA) and Y(ND) was observed in HT plants (Figure 3). 

### 3.3. Root Colonization and Spore Density

AMF enriched plants under normal conditions had higher root colonization (~75–80%) and the colonization decreased under high-temperature stress (~40–45%). The colonization in non-AM plants exposed to high temperatures was negligible. The spore count was significantly higher in the rhizosphere of AMF enriched plants (22–26 spores g^−1^ soil). In AMF + HT soil, the spore count was ~10–15 spores/g. No spores were detected in the non-AM plants grown in HT treatment. Control pots had 2.26 spores/g soil and the lowest root colonization (1.33%).

### 3.4. PLFA Analysis

Irrespective of the stress, AMF enrichment significantly enhanced 16:1ω5cis NLFA. A Significantly higher content was detected in AMF enriched plants (35.28 nanomoles NLFA g^−1^ soil) in comparison to the other treatments (Table 3). This was followed by AMF + HT plants under stress (20.66 nanomoles NLFA g^−1^ soil). Control plants and the plants under HT stress contained significantly lower 16:1ω5cis NLFA (10.78 nanomoles NLFA g^−1^ soil and 10.18 nanomoles NLFA g^−1^, respectively) than their AMF counterparts (Table 3). 

Significant differences were observed in the content of 16:1ω5cis PLFA across all the treatments examined. However, the content was highest in the pots under HT stress (6.21 nanomoles PLFA g^−1^). Remarkably, AMF enriched pots either under HT (4.97 nanomoles PLFA g^−1^) or without high temperature exposure (4.44 nanomoles PLFA g^−1^) contained lower PLFA than the control (5.96 nanomoles PLFA g^−1^) and HT pots (6.21 nanomoles PLFA g^−1^) (Table 3). 

The number of fungal biomarkers was statistically at par across all the treatments studied. Nevertheless, the content was the highest in the AMF enriched pots (1.63 nanomoles PLFA g^−1^ soil) followed by AMF + HT under stress (1.52 nanomoles PLFA g^−1^ soil). Irrespective of AMF application, the content of Gram-positive bacterial biomarker was higher in pots under HT stress (HT: 53.93 nanomoles PLFA g^−1^ soil, AMF + HT: 49.70 nanomoles PLFA g^−1^ soil). The population of actinomycetes was slightly higher in the pots devoid of AMF enrichment than AMF enriched pots (Control: 19.0 nanomoles PLFA g^−1^ soil, HT: 20.24 nanomoles PLFA g^−1^ soil) (Table 3). 

The Gram-negative bacterial biomarker followed the same trend as 16:1ω5cis PLFA. It was significantly higher in control pots (44.80 nanomoles PLFA g^−1^ soil). AMF enriched pots recorded a significantly lower Gram-negative bacterial population (34.21 nanomoles PLFA g^−1^ soil) than in the other treatments (Table 4).

The fungal to bacterial ratio differed non-significantly across all the treatments examined. The fungi/bacteria ratio was slightly higher in AMF + HT pots (0.0833) than in the other treatments. Similarly, the Gram-positive/Gram-negative ratio was also statistically at par across all the treatments tried.

### 3.5. MDA Content 

Total malondialdehyde (MDA) content decreased in AMF colonized plants compared to control plants. Maximum MDA content was observed in high temperature exposed plants, while AMF + HT plants showed a lower MDA content than HT maize plants (Table 2).

### 3.6. Principal Component Analysis (PCA)

**Figure 4:**
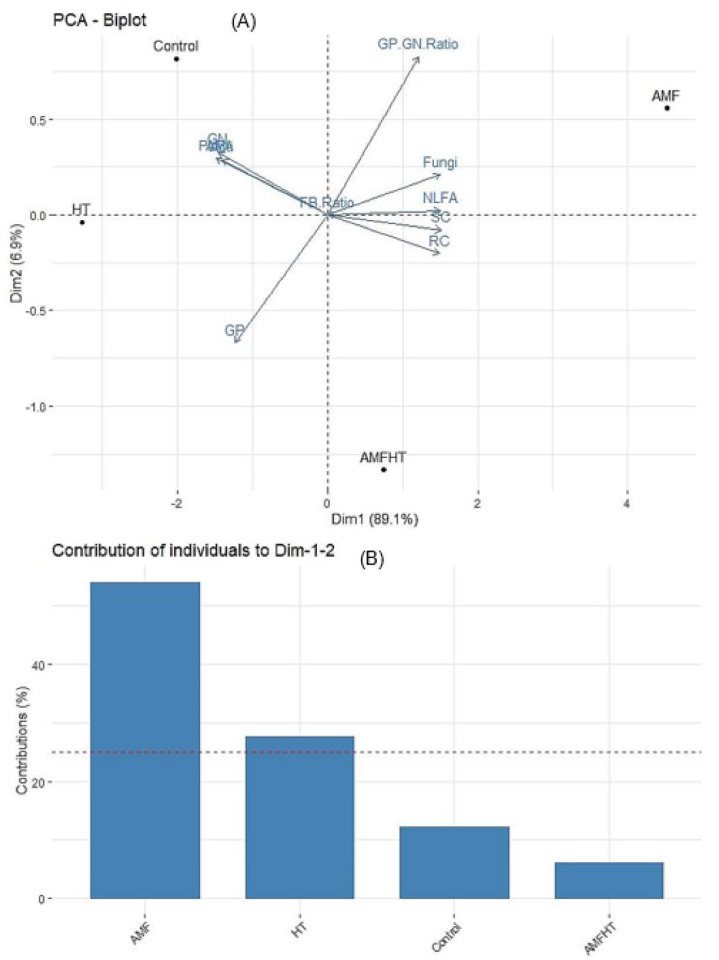
(**A**) PCA score plot for the treatments and parameters (AMF biomass and microbial communities). The percent values that indicate the variation contributed by each PC are displayed in parentheses. The highest contribution was made by the parameters present in the right (Table S1). (**B**) Graphical illustration of individual contribution of the treatments to PCs. Control = maize plants grown in normal soil. AMF = maize plants grown in AMF enriched soil, HT = maize plants grown in normal soil under higher temperature (natural temperature during summer 43 °C). AMF + HT = maize plants grown in AMF enriched soil under high-temperature stress. Fungi = fungal biomass, AM = PLFA = 16:1ω5cis (AM signature fatty acid biomarker for hyphal biomass), SC = spore count, RC = root colonization, NLFA = 16:1ω5cis neutral lipid fatty acid (AM signature fatty acid biomarker for storage lipids), GN = Gram-negative bacteria, GP GN ratio = Gram-positive/Gram-negative ratio, Acti = actinomycetes, GP = Gram-positive, FB ratio = Fungi/Bacteria ratio.

The result of PCA indicated the individual contribution of PLFA biomarkers to principal components. The score plots revealed that AMF played a critical role in directing the variation between treatments (Figure 4A, Table S1). The PC1 and PC2 described 95% (89.1% and 6.9%) to the total variation in the data where PC1 was heavily loaded with fungi (0.99), spore density (1.00), and root colonization (0.98), 16:1ω5cis NLFA (0.99), Gram-positive/Gram-negative ratio (0.80), whereas PC2 was loaded with the biomarkers for Gram-negative bacteria (0.22), 16:1ω5cis PLFA (0.19), and actinomycetes (0.19). The fungi/bacteria ratio had no variance among PCs (0.00) (Figure 4A, Table S1).

The most significant variation, which was detected in PC1 (89.1%), validated that AMF and non-AMF treatments have markedly different microbial signatures. Regarding the individual contribution of the treatments to the total variation (PC1 and PC2), the following trend was observed: HT > AMF > control > AMF + HT (Figure 4B).

The contribution of crucial physiological parameters was apparent in the PCA loading plot (Figure S1A). The distinction between physiological parameters was based mainly on PC1 (96.7%), which was loaded with YI (0.99), YII (1.00), ETRI (0.99), ETRII (0.99), P_m_ (0.98), q_L_ (0.99), q_P_ (0.98) and Fm (0.97). PC2 (2.6%) displayed less influence and was loaded with YNPQ (0.35), q_N_ (0.19), and YNA (0.01) (Figure S1A, Table S2).

The scores plot of the PCA analysis also revealed comprehensible groupings of the treatments (Figure S1A). The treatment groups were distinctly separated along PC1 and PC2. PC1 was loaded with AMF and control plants and PC2 with HT and AMF + HT plants (Figure S1A). Based on physiological parameters, the individual contribution of the treatments to the total variation (PC1 and PC2) was as follows: HT > AMF > control > AMF + HT (Figure S1B).

To further study the variation contributed by the parameters, PCA was applied with physiological parameters and signature lipids (Figure S2A). The first component of the PCA seemed to echo variances in P_m_ (1.00), q_L_ (0.98), ETRI (0.98), ETRII (0.98), YI (0.99), YII (0.96), Fm (0.96), q_P_ (0.95), fungi (0.88), Gram-positive/Gram-negative ratio (0.80) and 16:1ω5cis NLFA (0.80). The second component showed variances in 16:1ω5cis PLFA (0.59), actinomycetes (0.37), and Gram-negative (0.74) (Figure S2A, Table S3). Among the signature lipids, Gram-positive bacteria and 16:1ω5cis NLFA and among physiological parameters YNA, YND, YI, P_m_, and YNO made the highest contribution to the total variance accounted by PC1 and PC2 (Figure S2B).

## 4. Discussion

Various studies have reported that AMF improved stress mitigation and tolerance in plants [11,13,14,23,42,43]. AMF increases plant growth by improving nutrition acquisition, root architecture, and increasing antioxidant activity, as well as stress tolerance [44,45,46].

### 4.1. Total Chlorophyll Content

Higher chlorophyll content in AMF enriched plants compared to high temperature exposed plants could be associated with an increased photosynthesis rate or an increase in the N and Mg content (major components of chlorophyll molecules) of plants accompanied with an increased carbohydrate/sugar accumulation. In other words, AMF symbiosis creates a carbon sink in plants and consequently increases photosynthesis [13,23,47], which was evident in our study (Table 2). This subsequently led to higher production in photosynthates and biomass evident by higher Chl content in AMF and AMF + HT exposed maize plants. Higher chlorophyll content thus led to improved overall photosynthesis in AMF and AMF + HT plants which was confirmed by measuring Chl *a* fluorescence.

### 4.2. Chlorophyll a Fluorescence

In high temperature exposed plants, a decline in Y(II) indicates decreased quantum efficiency, as well as the closing of the open reaction centers. The major decrease in Y(II) is mainly due to a significant reduction in the fraction of open or active PSII reaction center. In the case of AMF + HT plants, AMF protected the plants by enhancing their photosynthetic efficiency. The decline in YII was accompanied by an increase in Y(NO) representing a significant inhibitory effect of HT on photochemical energy utilization of PSII. Higher non-regulated heat dissipation was a consequence of the PSII center being closed as electron transport was inhibited under HT exposure. It is speculated that the higher the fraction of close PSII centers, the higher will be the value for Y(NO). Y(NO) is also considered an excellent indicator of PSII damage. Y(NO) was found to be very low for AMF and control plants representing a healthy state of plants (Figure 1A). The declined quantum yield of PSII was also supported by reduced electron transport rate ETR_(II)_ (Figure 2A) and an increase in the value of Y(NO) indicating that both photochemical energy conversion and protective regulatory mechanisms were inefficient to protect the plant under HT stress. NPQ, one of the most efficient photoprotective responses and mechanisms, significantly increased under HT conditions. Since the phenomenon of NPQ takes place in the antenna system, it is an efficient protective reaction from overstimulation that could result in the formation of reactive oxygen species (ROS). It is suggested that the higher NPQ values are indicative of a more efficient energy dissipation mechanism that protects the photosynthetic apparatus of leaves from light-induced damage under HT conditions [48], which was indicated in our study. AMF enrichment could improve the energy dissipation ability of a plant, which protected the photosynthetic apparatus against excess light under HT exposure. AMF showed minimum NPQ values in maize plants, probably due to the increased utilization of photons (Figure 1A). The q_P_ values indicated that AMF enrichment can improve the utilization of photons and improve PSII susceptibility to photoinhibition and temperature stress. The coefficient of NPQ, q_N_ tells us about the proportion of the absorbed light energy being dissipated into heat. This indicates less efficiency of PSII in the use of excitation energy for photochemical reactions. q_N_ increased dramatically in HT exposed maize plants while it slightly increased in AMF + HT (Figure 1B). It was minimal in AMF plants depicting that the absorbed light energy was being utilized in photochemistry instead of getting dissipated as heat. A low value of q_L_ with HT exposure in maize plants reflected the over-reduction of PSII reaction centers. Increased q_L_ in AMF + HT indicated more number of active and open reaction centers due to the presence of AMF (Figure 1B). The inhibition of electron transport at any point in the whole electron transport chain creates an excitation pressure over PSII, which can be observed as a decrease in ETR_(II)_ and YII in HT exposed maize plants. A moderate change in ETR_(II)_ and ETR_(I)_ in AMF + HT plants was observed (Figure 2A). It is well known that increasing temperature increases thylakoid membrane fluidity [49]. The efficacy of electron transport can be hindered by high temperature induced structural changes in the protein complexes, downregulation of PSI and PSII, and inhibition of oxygen-evolving complex. These results suggest that electron transport after PSII was blocked, leading to excess energy production that could not be safely dissipated [50]. ETR_(I)_ was comparatively less affected indicating that PSI was a little bit robust as compared to PSII. The redox poise in the AMF maize plants was sustained even under HT conditions, which in turn, maintained the stable electron transport rate as evident by enhanced ETR_II_ and ETR_I_ rates in AMF + HT plants. P_m_ was measured through saturation pulse application with pre-illumination of far-red light. The measured decrease of P_m_ in HT exposed plants could also be due to either photoinactive PSI or due to decreased content of photo-oxidizable PSI per leaf area unit [51], while these values recovered in AMF + HT. A decline in F_m_ in HT plants suggests an imbalance in the photosynthetic apparatus leading to an increase in the possibility of dissociation of light-harvesting complex from PSII and thus disrupting the energetic connectivity [52]. This was recuperated in AMF + HT plants indicating that AMF protected the maize plants and photosynthesis was enhanced.

AMF maize plants showed maximum quantum yield for PSI (YI). AM fungi may have provided the host plants with additional transport channels for improving the uptake of water and nutrients from the soil through external hyphae [13,53] resulting in enhanced Y(I) (Figure 3). The low quantum yield of PSI in HT-exposed maize plants resulted from noticeable donor side limitation of PSI, as depicted by Y(ND), which was higher in HT exposed plants than in AMF plants. By these results, it can be interpreted that the fraction of the PSI complex having the capability of charge separation and stabilization reduced under HT treatment. In AMF + HT plants, the PSI complex was still in oxidizable form and PSI photochemistry was still possible. Y(ND) bumped up in HT plants due to inefficient light absorption by the antennae of PSII which provides electrons by water splitting and PSI. Ineffective absorption reduced the rates of PSII charge separation that did not match the capacity of PSI [27]. The small PSII antenna size can efficiently restrict PSI photochemistry via donor-side limitation. Decreased Y(I), also inhibited electron flow in the intersystem chain and lesser electrons coming from the stromal donors for PSI. The decrease in Y(I) is thus caused by an increase in the donor and acceptor side limitations of PSI. Moreover, when compared to control and AMF, the value of Y(NA) increased in HT exposed plants. The higher value of Y(NA) in HT plants demonstrated that high-temperature stress reduced the fraction of oxidized P700 because of lack of acceptors which may be explained as an adaptation to the donor-side limitation of PSI. Higher Y(NA) represented an over-reduction of the PSI acceptor side, which contributed to the photoinhibition of PSI, suggesting a shortage of electron acceptors (NADP^+^ or oxidized ferredoxin) resulting from low CO_2_ fixation [54]. It is assumed that the effect of HT exposure reduced the number of active reaction centers, while enrichment with AMF enhanced the number of active reaction centers [23]. AMF + HT plants were less affected by higher temperature as evident by the higher values of Y(I), Y(NA), and Y(ND) (Figure 3). The fluorescence studies were in accordance with the content of stress biomarkers in soil. Overall, AMF ameliorated the damaging effects of high-temperature stress by protecting PSI and PSII, enhancing photosynthesis, and providing proper nutrients and moisture from soil which further improved plant growth under stress conditions. AMF not only enhanced the photosynthesis but also protected the plants, which was in corroboration with the NLFA results as well.

### 4.3. Root Colonization and Spore Density

Although HT stress decreased root colonization in plants, AMF + HT plants had substantially higher colonization than HT exposed plants. It is speculated that AMF facilitated the high temperature exposed maize plants to protect them from temperature stress. In AMF + HT exposed maize plants, an increase in AMF biomass (spore density and root colonization) was observed as compared to control and HT plants. This indicated the strategy of AMF + HT plants to combat the stress caused by high temperature by allocating more C to AMF, thereby increasing the AMF biomass in soil. Previously, Hussain et al. [11] have reported that AMF coating on seeds enhanced root colonization in maize plants. AMF not only improves colonization and protects plants under high-temperature stress as shown in the present study but also mitigated the impact of Al toxicity in lotus and barley plants by accumulating Al in roots and ultimately protecting the plants from metal stress as well [14]. Root colonization helped in the upregulation of photosynthetic rate in AMF and AMF + HT plants. The results obtained from root colonization were studied in depth by the study of NLFA and PLFA in the presence of AMF under high temperature exposure in plants.

### 4.4. PLFA

It has been reported that the sensitivity of NLFA as an indicator of AMF biomass is more significant than 16:1ω5cis PLFA [55]. In our study, the effect of treatments on 16:1ω5cis NLFA was highly significant (Table 3 and Table 4). The abundance of 16:1ω5cis NLFA in AMF + HT over control and HT alone depict the plant mediated stress mitigation strategy which was also evident from fluorescence results. The 16:1ω5cis PLFA did not maintain consistency with NLFA and microscopic measures of AM biomass and a higher content was observed in control and HT. A similar trend observed in the case of Gram-negative and 16:1ω5cis PLFA is due to the presence of 16:1ω5cis PLFA in Gram-negative bacteria [56] and therefore the content of 16:1ω5cis PLFA may be higher in control and HT pots. Many studies have reported that as the soil gets warmed (due to HT), it encourages the proliferation of AMF extra radical hyphae [57] as a result of which the hyphae penetrate deep inside the soil and increases the supply of nutrients and moisture [58] which was evident in our study. This hyphal elongation further indicates an undeviating response of AMF under HT stress.

Temperature stands as a key environmental factor that impacts PLFA stress indicators [21]. The abundance of Gram-positive and actinomycete biomarkers at HT aligns with previous studies [21] where, as a consequence of warming, an increase in the relative abundance of Gram-positive and actinomycete biomarkers was observed. However, the population of actinomycete in the AMF + HT pots was lower possibly due to alleviation of stress by AMF. Moreover, Gram-positive bacteria increase at a higher temperature, which is related to decreased substrate availability and greater environmental stress resistance than Gram-negative bacteria [22], and therefore, the population of Gram-positive bacteria was higher in HT pots irrespective of AMF enrichment. It has been found that, in comparison to bacterial biomass, a higher temperature is more detrimental to fungal biomass [59]. Consistent with other studies [21], the population of fungi, was lowest in HT. However, this was not the case in AMF + HT, which is also attributable to AM-induced stress alleviation.

The higher ratio of fungi to bacteria ratio in AMF + HT plants indicates a less stressed scenario of soils resulting from higher AMF and fungal biomass. At higher temperatures, the adaptable microbial groups are selected from the microbial community and the less adjustable/tolerant ones are inhibited [60]. The inconsistency exhibited by the Gram-positive/Gram-negative ratio is in agreement with earlier studies, where the variation was credited to species succession instead of phenotypic adaptation to stress [60].

AMF and AMF + HT treatments were visibly distinguished from control and HT. Gram-negative/Gram-positive ratio, fungi, 16:1ω5cis NLFA, spore count, and root colonization were positively correlated with each other and were present with AMF and AMF + HT. 16:1ω5cis NLFA depicts AMF storage lipids viz., spores, and vesicles [55] and this substantiates the significant and positive correlation observed between 16:1ω5cis NLFA and spore density. Additionally, the positive correlation between 16:1ω5cis NLFA and root colonization can be attributed to the presence of AMF storage structure inside the plant roots which is a characteristic of *Glomus* species. The variables viz., Gram-negative, actinomycetes, 16:1ω5cis PLFA, Gram-positive, were present along PC2 with control and HT. The negative correlation of 16:1ω5cis PLFA with NLFA and microscopic parameters might have arisen from the 16:1ω5cis PLFA of Gram-negative bacterial origin [56].

In the case of physiological parameters, Fm, ETRI, ETRII, YI, YII and q_p_ were present with AMF and control while, YNPQ, q_N_, YNA, YNO, YND, P_m_, and q_L_ were present along with AMF + HT and HT. Microbial membranes are more subtle to environmental perturbations, and therefore, the response of fatty acid biomarkers to the treatment-induced effects was quite spontaneous. However, NLFA 16:15cis and microscopic parameters were significantly and positively correlated with photosynthetic parameters (YII, YI, ETRI, ETR_II_, P_m_, F_m_, q_L_, q_P_). Similarly, a significant correlation of photosynthetic parameters (YII, YI, ETRI, ETRII, P_m_, F_m_, q_L_, q_P_) with the Gram-positive/Gram-negative ratio was observed.

### 4.5. MDA Content

After validating the effect of high-temperature stress on physiological parameters and soil quality indicators, and to ensure its effect on membrane integrity, MDA content was measured. In general, malondialdehyde (MDA) is coupled with the peroxidation of polyunsaturated fatty acids in the membrane and subsequently with cellular integrity. Lipid peroxidation indicates oxidative tissue damage by hydrogen peroxide, superoxide, hydroxyl radicals. This results in the structural alteration of membranes with the release of cell and organelle content, loss of essential fatty acids, and formation of cytosolic aldehyde and peroxide products [61]. ROS react with lipids leading to the formation of highly active peroxy radicals, which in turn starts a cascade reaction. The level of MDA decides the degree of membrane lipid peroxidation [61]. Damage to cell membrane indicated by higher concentrations of MDA content was observed in HT exposed maize plants when compared with control, AMF, and AMF + HT plants (Table 2), which is also evident from the results obtained from photosynthesis measurements. Temperature stress caused the peroxidation of membrane lipids, however in AMF + HT plants, MDA content decreased due to the presence of AMF which protected the plants. Our results are in accordance with the previous studies where AM symbiosis improved plant defense against HT stress by decreasing the level of lipid peroxidation (MDA) [62] and improving photosynthesis. Several studies have also demonstrated that MDA content in AM plants was lower than that in the non-AM plants [62]. A similar trend was observed in our study, which indicates that AM symbiosis could alleviate the peroxidation of membrane lipids and maintain the fluidity of the membrane [63]. This suggests that AMF not only protected the cell membrane from ROS damage but also improved photosynthesis.

## 5. Conclusions

We put forward the evidence that under high temperature exposure, arbuscular mycorrhizal fungi symbiosis (AMF enrichment) can facilitate high photosynthetic capacity and prevent the photosynthetic apparatus from being damaged. The damaging effect of high-temperature stress on PSI and PSII was restrained by arbuscular mycorrhizal fungi enrichment. AMF + HT plants showed recovery for high temperature exposure maize plants for all of the parameters studied. In support of the physiological parameters (evident from fluorescence results), the abundance of NLFA in AMF + HT over control and high temperature exposed plants alone indicate the arbuscular mycorrhizal mediated stress mitigation strategy to protect itself and survive under high temperature exposure. Furthermore, our hypothesis concludes that not only the above-ground but the below-ground parameters, with higher content of NLFA and ratio of Gram-positive to Gram-negative bacteria in stressed plants could be one of the probable indications that arbuscular mycorrhizal fungi helped the plants in ameliorating high-temperature stress. Taken together, these results indicate that arbuscular mycorrhizal fungi help the plant to maintain the stability of PSI and PSII, improves the damaging effect of high temperature exposure, enhanced photosynthesis, soil quality, and crop growth leading to improvements in the yield. This is the first study combining plant physiological traits (aboveground) and PLFA (belowground) parameters bringing novel insight into plant improvement under stress conditions.

## Figures and Tables

**Table 1:** Description of various Chlorophyll *a* fluorescence parameters [28,29,30,31,32].

Fluorescence Parameters	Description
ETR	electron transport rate
F_o_	minimal fluorescence
F_m_	maximum fluorescence
P_m_	maximal change in P700 signal during the quantitative transformation of P700 from the fully reduced to the fully oxidized state
q_L_	fraction of open PSII reaction centers
q_P_	photochemical quenching coefficient used to assess PSII susceptibility to photo-inhibition and reflects the oxidation-reduction state of the primary acceptor (Q_A_) for PSII
q_N_	non-photochemical quenching coefficient, i.e., the fraction of dark-adapted variable fluorescence that is lost upon adaptation to light
Y(I)	effective photochemical quantum yield of PSI
Y(II)	effective quantum yield of PSII
Y(NA)	quantum yield of non-photochemical energy dissipation of reaction centers due to PSI acceptor-side limitation
Y(ND)	quantum yield of non-photochemical energy dissipation in reaction centers due to PSI donor-side limitation
Y(NO)	quantum yield of non-regulated energy dissipation and the fraction of energy that is passively dissipated in the form of heat and fluorescence mainly due to the closed PSII centers
Y(NPQ)	quantum yield of light-induced non-photochemical fluorescence quenching

**Table 2:** Malondialdehyde (MDA) content (μM mg^−^^1^ FWL) and total chlorophyll content in maize plants.

Treatments	MDA Concentration (μM mg^−1^ FWL)	Total Chl Concentration (SPAD Units)
Control	57.0 ± 2.01	41 ± 1
AMF	32.0 * ± 2.0	46 * ± 1
HT	167 *** ± 10.06	20 *** ± 1
AMF + HT	88.0 ** ± 4.01	37 ** ± 2

The data are the mean values of three replicates ± standard deviation. Significance was determined according to Dunnet comparison of all columns versus control column at *p* < 0.01 (* *p* < 0.05, ** *p* < 0.01, and *** *p* < 0.001).

**Table 3:** The effect of high-temperature stress on PLFA communities (nanomoles g^−1^) analyzed in maize rhizosphere.

Treatment	16:1ω5cis PLFA	16:1ω5cis NLFA	Gram-Negative	Gram-Positive	Fungi	Actinomycetes
Control	5.96 ± 0.42 ^a^	10.78 ± 5.17 ^c^	44.80 ± 4.18 ^a^	47.30 ± 16.95 ^a^	1.48 ± 0.35 ^a^	19.00 ± 5.82 ^a^
AMF	4.44 ± 0.01 ^c^	35.28 ± 0.64 ^a^	34.21 ± 3.54 ^b^	43.77 ± 4.59 ^a^	1.63 ± 0.00 ^a^	17.30 ± 1.62 ^a^
HT	6.21 ± 0.06 ^a^	10.18 ± 0.00 ^c^	43.50 ± 2.50 ^ab^	53.93 ± 3.88 ^a^	1.45 ± 0.26 ^a^	20.24 ± 3.53 ^a^
AMF + HT	4.97 ± 0.32 ^b^	20.66 ± 1.54 ^b^	38.83 ± 5.04 ^ab^	49.70 ± 6.89 ^a^	1.52 ± 0.01 ^a^	17.71 ± 3.19 ^a^
LSD (*p* = 0.05)	0.50	5.11	7.39	18.13	0.41	7.23
Main effect	***	***	*	ns	ns	ns

The data are the mean values of three replicates ± standard deviation, treatment means followed with different letters vary significantly at *p* = 0.05 in compliance with Fisher least significant differences (LSD) and Duncan multiple range test (DMRT) for multiple comparisons. AM = 16:1ω5cis PLFA (AM signature fatty acid biomarker for hyphal biomass), NLFA= 16:1ω5cis neutral lipid fatty acid (AM signature fatty acid biomarker for storage lipids). Significance was determined at *p* < 0.01 (ns = non-significant, * *p* < 0.05, and *** *p* < 0.001) and the results are expressed as mean values and standard deviation (SD).

**Table 4:** The effect of high-temperature stress on fungi/bacteria-specific PLFA community analyzed in maize rhizosphere.

Treatment	Fungi/Bacteria	Gram-Positive/Gram-Negative
Control	0.0767 ± 0.01 ^a^	1.52 ± 0.09 ^a^
AMF	0.0800 ± 0.01 ^a^	1.60 ± 0.03 ^a^
HT	0.0800 ± 0.01 ^a^	1.51 ± 0.08 ^a^
AMF + HT	0.0833 ± 0.01 ^a^	1.50 ± 0.09 ^a^
LSD (*p* = 0.05)	0.02	0.14
Main effect	ns	ns

The data are the mean values of three replicates ± standard deviation, treatment means followed with different letters vary significantly at *p* = 0.05 in compliance with Fisher least significant differences (LSD) and Duncan multiple range test (DMRT) for multiple comparisons. Significance was determined at *p* < 0.01 (ns= non-significant) and the results are expressed as mean values and standard deviation (SD).

## Data Availability

All data supporting the findings of this study are available within the paper and within its Supplementary Materials published online.

## Supplementary Materials

The following are available online at https://www.mdpi.com/article/10.3390/jof7100867/s1, Figure S1: (A) PCA score plot for the treatments and physiological parameters. The percent values that indicate the variation contributed by each PC are displayed in parentheses. The highest contribution was made by the parameters present in the right (Supplementary Table S2). (B) Graphical illustration of individual contribution of the treatments to PCs. Control = maize plants grown in normal soil. AMF = maize plants grown in AMF enriched soil, HT = maize plants grown in normal soil under higher temperature (natural temperature during summer 43 °C). AMF + HT = maize plants grown in AMF enriched soil under high-temperature stress. Pm = Response of maximal change in P700, F_m_ = maximum fluorescence, ETR_I_ = relative electron transport rates in PSI, ETR_II_ = PSII with the application of a saturation pulse, qp = photochemical quenching, qn = non-photochemical quenching coefficient, q_L_ = fraction of open PSII reaction centers, Y(I) = quantum yield of PSI, Y(II) = quantum yield of PSII, Y(NA) = quantum yield of non-photochemical energy dissipation due to acceptor-side limitation, Y(ND) = quantum yield of non-photochemical energy dissipation due to donor-side limitation, Y(NO) = yield of non-regulated energy dissipation, Y(NPQ) = yield of regulated energy dissipation. Figure S2: (A) PCA score plot for the physiological parameters and signature lipids. The percent values that indicate the variation contributed by each PC are displayed in parentheses. The highest contribution was made by the parameters present in the right (Supplementary Table S3). (B) Graphical illustration of individual contribution of the physiological parameters and signature lipids to PCs. P_m_ = Response of maximal change in P700, F_m_ = maximum fluorescence, ETR_I_ = relative electron transport rates in PSI, ETR_II_ = PSII with the application of a saturation pulse, qp = photochemical quenching, q_N_ = non-photochemical quenching coefficient, qL = fraction of open PSII reaction centers, Y(I) = quantum yield of PSI, Y(II) = quantum yield of PSII, Y(NA) = quantum yield of non-photochemical energy dissipation due to acceptor-side limitation, Y(ND) = quantum yield of non-photochemical energy dissipation due to donor-side limitation, Y(NO) = yield of non-regulated energy dissipation, Y(NPQ)= yield of regulated energy dissipation, fungi= fungal biomass, AM = PLFA = 16:1ω5cis (AM signature fatty acid biomarker for hyphal biomass), NLFA = 16:1ω5cis neutral lipid fatty acid (AM signature fatty acid biomarker for storage lipids), GN = Gram-negative bacteria, GP GN ratio = Gram-positive/Gram-negative ratio, Acti = actinomycetes, GP = Gram-positive, FB ratio = Fungi/Bacteria ratio. Table S1: Eigen values, variance, and variable coordinates of different signature lipids, and AM associated microscopic variables assessed in the soil corresponding to Figure 4A (Principle component analysis score plot) PCA loadings N > 0.5 are shown in bold. Table S2: Eigen values, variance, and variable coordinates of different physiological variables assessed in plants corresponding to Figure S1A (Principal component analysis score plot) PCA loadings N > 0.5 are shown in bold. Table S3: Eigen values, variance, and variable coordinates of different physiological variables and signature lipids assessed in plants corresponding to Figure S2A (Principle component analysis score plot).

## Abbreviations

(AMF), Arbuscular mycorrhizal fungi; (Chl), Chlorophyll; (ETR), electron transport rate; (HT), high temperature; (NLFA), neutral lipid fatty acid; (PLFA), phospholipid fatty acid; (q_p_), photochemical quenching; (ROS), reactive oxygen species; (TBARS), thiobarbituric acid reactive substances; Y(I), quantum yield of PSI; Y(II), quantum yield of PSII; Y(NA), quantum yield of non-photochemical energy dissipation due to acceptor-side limitation; Y(ND), quantum yield of non-photochemical energy dissipation due to donor-side limitation; Y(NO), yield of non-regulated energy dissipation; Y(NPQ), yield of regulated energy dissipation.
