# Supplementary material for: Effect of High-Temperature Stress on Plant Physiological Traits and Mycorrhizal Symbiosis in Maize Plants
Source: J Fungi (Basel). 2021 Oct 16;7(10):867. doi: 10.3390/jof7100867 (PMC8539748; doi:10.3390/jof7100867)
Supplement: Supplementary file 1 [file jof-07-00867-s001.zip › jof-1377432-supplementary.pdf]

Supplementary Materials

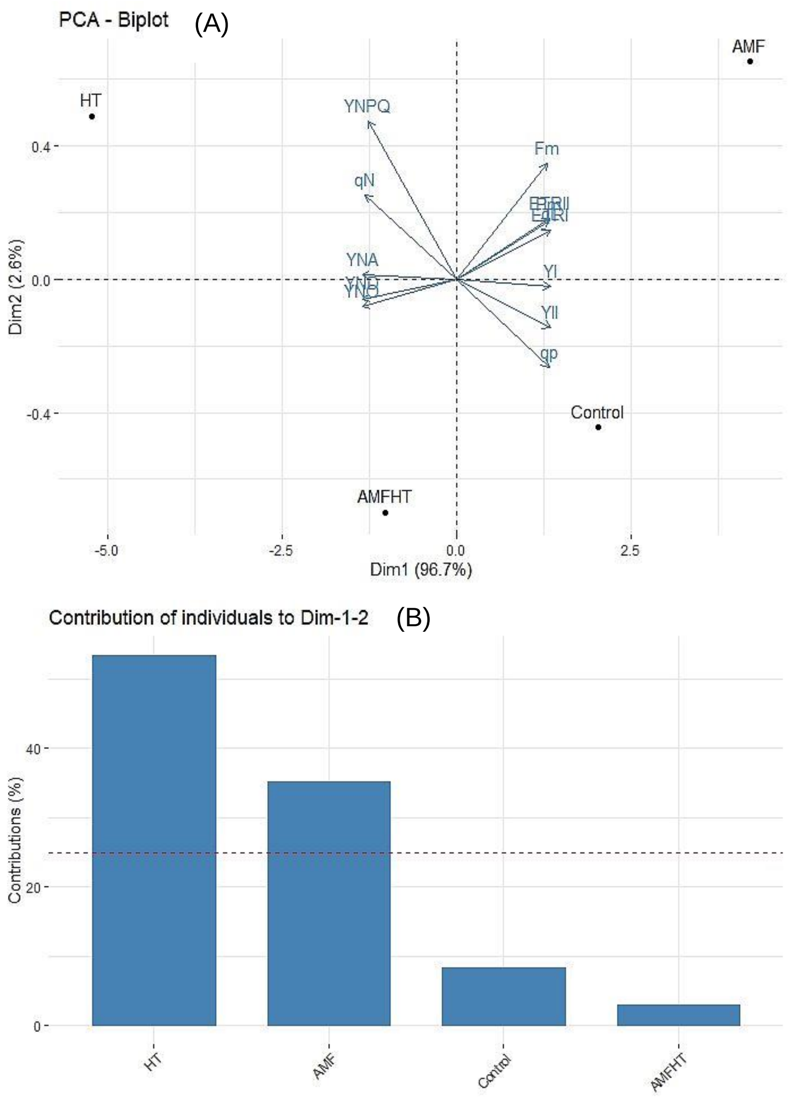

Figure S1(A) PCA score plot for the treatments and physiological parameters. The percent values that indicate the variation contributed by each PC is displayed by parenthesis. The highest contribution was made by the parameters present in the right (Supplementary Table S2). **(B)** Graphical illustration of individual contribution of the treatments to PCs. Control = maize plants grown in normal soil. AMF = maize plants grown in AMF enriched soil, HT = maize plants grown in normal soil under higher temperature (natural temperature during summer 43 °C). AMF+HT = maize plants grown in AMF enriched soil under high temperature stress. Pm= Response of maximal change in P700, F_m= maximum fluorescence, ETR_I= relative electron transport rates in PSI, ETR_{II} =PSII with the application of a saturation pulse, qp= photochemical quenching, qn= non-photochemical quenching coefficient, qL= fraction of open PSII reaction centres, Y(I)= quantum yield of PSI, Y(II)= quantum yield of PSII, Y(NA)= quantum yield of non-photochemical energy dissipation due to acceptor-side limitation, Y(ND)= quantum yield of non-photochemical energy dissipation due to donor-side limitation, Y(NO)= yield of non-regulated energy dissipation, Y(NPQ)= yield of regulated energy dissipation.

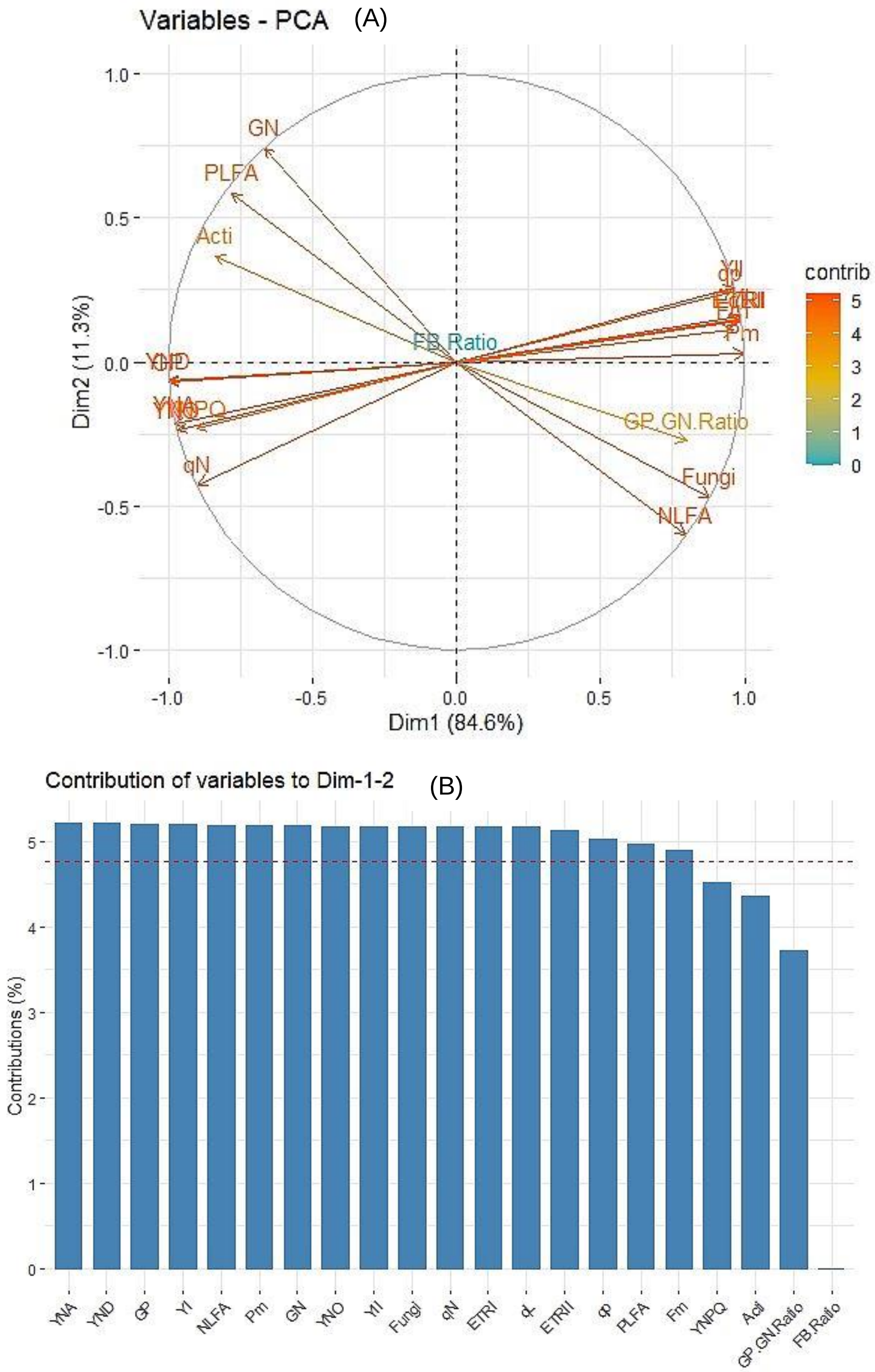

Figure S2(A) PCA score plot for the physiological parameters and signature lipids. The percent values that indicate the variation contributed by each PC is displayed by parenthesis. The highest contribution was made by the parameters present in the right (Supplementary Table S3). **(B)** Graphical illustration of individual contribution of the physiological parameters and signature lipids to PCs. Pm= Response of maximal change in P700, F_m= maximum fluorescence, ETR_I= relative electron transport rates in PSI, ETR_{II} =PSII with the application of a saturation pulse, qp= photochemical quenching, qn= non-photochemical quenching coefficient, qL= fraction of open PSII reaction centres, Y(I)= quantum yield of PSI, Y(II)= quantum yield of PSII, Y(NA)= quantum yield of non-photochemical energy dissipation due to acceptor-side limitation, Y(ND)= quantum yield of non-photochemical energy dissipation due to donor-side limitation, Y(NO)= yield of non-regulated energy dissipation, Y(NPQ)= yield of regulated energy dissipation, fungi= fungal biomass, AM= PLFA = 16:1ω5cis (AM signature fatty acid biomarker for hyphal biomass), NLFA= 16:1ω5cis neutral lipid fatty acid (AM signature fatty acid biomarker for storage lipids), GN= Gram-negative bacteria, GP GN ratio= Gram-positive/Gram-negative ratio, Acti= actinomycetes, GP= Gram-positive, FB ratio= Fungi/Bacteria ratio.

Table S1

Eigen values, variance and variable coordinates of different signature lipids and AM associated microscopic variables assessed in the soil corresponding to Figure 4A (Principle component analysis score plot) PCA loadings N > 0.5 are shown in bold.

**Factors**	**PC1**	**PC2**	**PC3**
Eigen value	8.91	0.69	0.41
% Total variance	89.08	6.90	4.02
% Cumulative variance	89.08	95.98	100
Variable coordinates			
Spore density	**1.00**	-0.05	0.06
Root colonization (RC)	**0.98**	-0.13	0.11
16:1ω5cis PLFA	-0.98	0.19	0.05
16:1ω5cis NLFA	0.99	0.01	0.14
Gram negative	-0.96	0.22	-0.19
Gram positive	-0.81	-0.44	0.38
Fungi	**0.99**	0.14	0.04
Actinomycetes	-0.92	0.19	0.34
Fungi/bacteria ratio	0.00	0.00	0.00
Gram negative/Gram positive	**0.80**	**0.54**	0.25

Table S2

Eigen values, variance and variable coordinates of different physiological variables assessed in plants corresponding to Figure S1A (Principle component analysis score plot) PCA loadings N> 0.5 are shown in bold.

**Factors**	**PC1**	**PC2**	**PC3**
Eigen value	12.58	0.34	0.08
% Total variance	96.75	2.60	0.65
% Cumulative variance	96.75	99.35	100
Variable coordinates			
YII	**0.99**	-0.11	-0.03
YNO	-0.99	-0.06	0.08
YNPQ	-0.93	0.35	-0.10
YI	**1.00**	-0.02	0.04
YNA	-1.00	0.01	0.03
YND	-0.99	-0.04	-0.12
ETRI	**0.99**	0.11	0.00
ETRII	**0.99**	0.13	-0.02
Pm	**0.98**	0.13	0.11
Fm	**0.97**	0.26	-0.04
qL	**0.99**	0.11	-0.01
qp	**0.98**	-0.19	0.03
qN	**-0.96**	0.19	0.19

Table S3
Eigen values, variance and variable coordinates of different physiological variables and signature lipids assessed in plants corresponding to Figure S2A (Principle component analysis score plot).

**Factors**	**PC1**	**PC2**	**PC3**
Eigen value	16.92	2.26	0.81
% Total variance	84.61	11.32	4.07
% Cumulative variance	84.61	95.93	100.00
Variable coordinates			
16:1ω5cis PLFA	-0.78	**0.59**	0.22
16:1ω5cis NLFA	**0.80**	-0.60	0.07
Gram negative	-0.67	**0.74**	0.07
Gram positive	-1.00	-0.07	-0.04
Fungi	**0.88**	-0.47	0.09
Actinomycetes	-0.84	0.37	0.41
Fungi/bacteria ratio	0.00	0.00	0.00
Gram negative/Gram positive	**0.80**	-0.28	0.54
YII	**0.96**	0.26	-0.09
YNO	-0.97	-0.24	-0.09
YNPQ	-0.90	-0.23	0.36
YI	**0.99**	0.16	-0.04
YNA	-0.98	-0.22	0.00
YND	-1.00	-0.07	0.01
ETRI	**0.98**	0.15	0.10
ETRII	**0.98**	0.15	0.13
Pm	**1.00**	0.03	0.07
Fm	**0.96**	0.11	0.25
qL	**0.98**	0.15	0.10
qp	**0.95**	0.24	-0.19
qN	-0.90	-0.43	0.10
